# Embedding comprehensive smoking cessation programs into community clinics: study protocol for a cluster-randomized controlled trial

**DOI:** 10.1186/s13063-022-06023-3

**Published:** 2022-02-03

**Authors:** Wave-Ananda Baskerville, Theodore C. Friedman, Brian Hurley, Susan Hsieh, Tasha Dixon, Norma Mtume, Martin L. Lee, Luz Rodriguez, Briana Lopez, Lara A. Ray

**Affiliations:** 1 Department of Psychology, University of California, Los Angles, Los Angeles, CA USA; 2 Department of Internal Medicine, Charles R. Drew University, Los Angeles, CA USA; 3 Los Angeles County Department of Health Services, Los Angeles, CA USA; 4 Friends Research Institute, Cerritos, CA USA; 5 David Geffen School of Medicine, University of California, Los Angeles, Los Angeles, CA USA; 6 Harbor-University of California, Los Angeles Medical Center, Los Angeles, CA USA; 7 Brain Research Institute, University of California, Los Angeles, Los Angeles, CA USA; 8 Department of Psychiatry and Biobehavioral Sciences, University of California, Los Angeles, Los Angeles, CA USA

**Keywords:** Smoking, Community clinics, Smoking cessation programs, Treatment, Cigarette use

## Abstract

**Background:**

Cigarette smoking among adults in the USA is a leading cause of preventable death worldwide, even though there has been a decline in prevalence since 2005. The addictive nature of nicotine is the chief reason smokers continue to use tobacco. Although the majority of smokers report a desire to quit smoking, a small minority who attempt to quit achieve long-term cessation. Combined, smoking cessation best practices include coordinated medication and behavioral treatments. However, these treatments are not currently adequately delivered to Medi-Cal beneficiaries in the publicly funded patient-centered medical homes (PCMHs) and community mental health clinics operated by Los Angeles County (LAC)-Department of Health Services (LACDHS) and LAC-Department of Mental Health (LACDMH).

**Methods:**

This is a 5-year implementation, cluster-randomized comparative effectiveness trial that will support the implementation of smoking cessation services delivered in LAC-LACDHS-operated outpatient primary care clinics and in LAC-LACDMH-operated community mental health clinics. We will enroll 1000 participants from clinics that will offer smoking cessation services and 200 from clinics that will offer treatment as usual. Participants will be asked to complete assessments at baseline, 3 months, 6 months, and 12 months. The assessments will include self-reports on smoking history, anxiety, stress, quality of life, and participant satisfaction. Participants who are assigned to clinics that provide smoking cessation services will also be asked about the frequency of their participation in the smoking cessation services during the 12-month period.

**Discussion:**

This study will evaluate the effectiveness and feasibility of implementing smoking cessation services in outpatient primary care and community mental health clinics. It will also determine if there will be higher rates of smoking cessation in the implementation sites as compared to the sites with treatment as usual. If the implementation proves to be effective, the plan is to sustain these services using a workflow we will develop in the LAC-operated sites. This would lead to ameliorating the significant smoking cessation treatment gaps among those served within the LAC Health Agency departments.

**Trial registration:**

ClinicalTrials.gov NCT04717544 “Embedding comprehensive smoking cessation programs into community clinics.” Registered on January 22, 2021

**Supplementary Information:**

The online version contains supplementary material available at 10.1186/s13063-022-06023-3.

## Administrative information

Note: the numbers in curly brackets in this protocol refer to SPIRIT checklist item numbers. The order of the items has been modified to group similar items (see http://www.equator-network.org/reporting-guidelines/spirit-2013-statement-defining-standard-protocol-items-for-clinical-trials/).

**Table:** 

Title {1}	Embedding comprehensive smoking cessation programs into community clinics
Trial registration {2a and 2b}.	ClinicalTrials.gov, NCT04717544 “Embedding comprehensive smoking cessation programs into community clinics”, registered January 22, 2021.
Protocol version {3}	Protocol version 7, created on January 16, 2021.
Funding {4}	This study is funded by the University of California’s Tobacco Related Disease Research Program (TRDRP) and the University of California Office of the President. Grant #: 28CP-0040.
Author details {5a}	Department of Psychology, University of California, Los Angles, Los Angeles, CA (WAB, LAR) Brain Research Institute, University of California, Los Angeles, Los Angeles, CA (LAR) Department of Psychiatry and Biobehavioral Sciences, University of California, Los Angeles, Los Angeles, CA (LAR) Department of Internal Medicine, Charles R. Drew University, Los Angeles, CA (TCF, MLL) Los Angeles County Department of Health Services, Los Angeles, CA (BH, TCF) Friends Research Institute, Cerritos, CA (TCF, TD, LR, BL, BH) David Geffen School of Medicine, University of California, Los Angeles, Los Angeles, CA (SH) Harbor-University of California, Los Angeles Medical Center, Los Angeles, CA (SH)
Name and contact information for the trial sponsor {5b}	University of California’s Tobacco Related Disease Research Program (TRDRP), Norval J. Hickman III, Ph.D., M.P.H., TRDRP Program Officer, Social & Behavioral Sciences <Norval.Hickman@ucop.edu>
Role of sponsor {5c}	The study sponsor/funder will have no role in collection, management, analysis, and interpretation of data; writing of the report; and the decision to submit the report for publication. The study sponsor did request minor changes in the study design based on the comments from the grant reviewers.

## Introduction

### Background and rationale {6a}

Tobacco use disorder is the leading cause of preventable death worldwide and is undertreated in the public sector. In 2018, the prevalence of current cigarette smoking among adults was 13.7% that was a significant decline from 2005 (20.9%) [1]. Additionally, cigarette use places an enormous burden on the US economy. From 2009 to 2012, smoking cost the USA between $289 and 332.5 billion (over that 3-year period) and between 46 and 53% of this is spent on adult medical care, while the rest is due to loss of productivity [2].

The prevalence of cigarette smoking is highest among adults who are male; aged 25–64 years, are American Indian/Alaska Native or multiracial; live below the federal poverty level; live in the Midwest or South; have a General Education Development (GED) certificate; are uninsured or insured through Medicaid; have a disability/limitation; are lesbian, gay, or bisexual; or have serious psychological distress [3]. Individuals with mental health and addictive disorders (MHAD) have higher rates of tobacco smoking and low rates of long-term smoking cessation, resulting in morbidity and mortality due to tobacco dependence [4, 5]. Barriers to treatment, such as inaccessibility to smoking cessation programs and medications, inhibit smoking cessation among those with comorbid MHAD and tobacco dependence [6]. Population-based interventions are critical to reducing the health and economic burden of smoking-related diseases among US adults, particularly among subpopulations with the highest prevalence of smokers.

The addictive nature of nicotine is the chief reason smokers continue to use tobacco. While about 70% of both adolescent and adult smokers state that they would like to quit smoking, only about 7% of smokers who try to quit on their own achieve long-term cessation each year [6]. There are a variety of treatment methods used to promote smoking cessation. A Cochrane database systematic review identified 12 treatment-specific reviews using pharmacological interventions for smoking cessation of which their analyses covered 267 studies, involving 101,804 participants [7]. The main outcome analyzed was smoking cessation although smoking reduction was also assessed. Some of the findings include that both nicotine replacement therapy (NRT) and bupropion were superior to placebo. Varenicline increased the odds of quitting compared with placebo. Head-to-head comparisons between bupropion and NRT showed equal efficacy. Varenicline was superior to single forms of NRT and to bupropion. Varenicline was more effective than nicotine patch, nicotine gum, and other NRT (inhaler, spray, tablets, lozenges), but was not more effective than combination NRT. Combined smoking cessation best practices include coordinated medication and behavioral treatments [2, 8, 9]. However, these treatments are not currently adequately delivered to Medi-Cal beneficiaries in the publicly funded PCMHs and community mental health clinics operated by LACDHS and LACDMH, respectively. With Los Angeles County being the second largest municipal health system in the nation, LACDHS and LACDMH serve 850,000 people annually. Of this population, 13% have tobacco use disorder (TUD) and tobacco-related disease attributes to one in seven deaths [10].

For these reasons, it is important to set up a comprehensive smoking cessation program in PCMHs and DMH clinics that will offer group counseling as well as medications for smoking cessation. We will conduct an implementation study of smoking cessation services delivered in LACDHS and LACDMH clinics lasting at least 5 years to determine if there will be higher rates of smoking cessation in the implementation sites compared to matched LAC-operated sites with treatment as usual (TAU).

### Objectives {7}

The aim of the present study is to create sustainable improved tobacco screening, treatment intervention, and cessation in LACDHS-operated outpatient primary care clinics and in LACDMH-operated community mental health clinics. This project will support the implementation of smoking cessation services delivered in LACDHS and LACDMH clinics and will evaluate the effectiveness and feasibility of these services. The primary outcome is to determine if there will be higher rates of smoking cessation in the implementation sites as compared with matched LAC-operated sites with TAU. Exploratory analysis will consider intervention effects on the smoking rate, such as smoking reductions. The primary aims of the current study are detailed below.

#### Primary aims

*Primary aim 1*: To test whether STUD participants have greater improvement in an objective measure of smoking behavior than TAU participants.

*Primary aim 2*: To test the penetrance of smoking cessation medications and counseling services in the STUDS arm vs. the TAU arm and between mental health and primary care clinics.

*Primary aim 3*: To test the dose effect on smoking cessation among those who receive medication and counseling services. Specifically, investigate if multiple medications plus counseling are more effective than single medication plus counseling, and what the dose-response effect of the number of counseling sessions on smoking abstinence.

## Trial design {8}

The study design consists of a 5-year implementation, cluster-randomized comparative effectiveness trial comparing intervention clinics with TAU. Intervention clinics provide smoking cessation group counseling and medication management and are integrated into primary care and community mental health clinics. TAU offers information about the California Smoker’s Helpline and usual provider and clinician counseling. Participants in TAU will be asked at each data collection point (baseline, 3, 6, and 12 months) about any smoking cessation services received including prescribed and over-the-counter smoking cessation medication. Each clinical site in LACDHS is part of a cluster, and there will be a 5:1 ratio in the assignment of clinics assigned to the intervention as compared with the TAU. There are eighteen outpatient clinics participating in the study, eleven from LACDHS and seven from LACDMH (see the “Study setting {9}” section for full details of study sites). The present study aims to enroll 1000 participants from the clinics that will offer smoking cessation services and 200 from clinics not offering smoking cessation services. There are four data collection points, baseline, 3 months, 6 months, and 12 months. Each data collection point will take approximately 30–45 min. These interviews will take place at the participant’s primary care clinic or the site associated with where they usually obtain county-provided services.

In the first quarter of year 1, the research team will revise and prepare study material which will include the participant consent forms, participant and clinical staff questionnaires, recruitment documents, and any other related study material. In the second quarter of year 1, year 2, and the first quarter of year 3, the research team will support the implementation of smoking cessation services by providing training to the clinical staff at the intervention site. The research team will meet with the staff at the intervention sites in quarters 2 and 3 of year 1 and quarter 3 of year 2. The intervention will begin in quarter 2 of year 1 and continue through quarter 4 of year 3. The project may continue recruitment if funding remains available.

At the first interview, the researcher will explain the study and answer any questions participants may have. If they agree to participate in the study, they will be enrolled into the study at the first interview. The researcher will then set up a schedule to meet again at 3 months, 6 months, and 12 months. To enhance retention, participants will be asked to provide contact information for the research team to keep in contact for subsequent follow-up interventions.

At each interview, the researcher will complete brief medical and mental health assessments which will ask about participants’ overall physical and mental health, including their smoking status. Data will be collected in-person by the research team via computer-assisted interviews which will include self-reports on smoking history, anxiety, stress, quality of life, and participant satisfaction. Initially, at each interview, we planned on asking participants to complete the carbon monoxide (CO) breathalyzer test and provide a urine sample for the rapid dipstick cotinine test to determine the use of cigarettes and levels of nicotine in the body. The urine collected for this study was to be only tested for cotinine and no other substances. Breathalyzer and urine testing were temporarily suspended in March 2020 in response to the COVID-19 pandemic when enrollment and follow-up assessments shifted from in-person to telephone and in-person visits are not anticipated to resume. Prior to suspension, 12 participants in the TAU arm and 69 participants in the intervention arm completed CO and urine testing.

Participants who are assigned to clinics that provide smoking cessation services will also be asked about the frequency of their participation in the smoking cessation services during the 12-month period. Interviews will be conducted at the participating LAC outpatient clinic where participants receive their primary health care. The smoking cessation services will include a (1) weekly 60–75-min cognitive-behavioral therapy (CBT) smoking cessation counseling group which will be led by facilitators that have been trained through the Kick Ash program and may be a nurse, pharmacist, clinician, substance use disorder (SUD) counselor, behavioral health clinician, or counselor; and (2) smoking cessation medication that will be prescribed by a licensed, prescribing clinician. Smoking cessation medications will include varenicline (Chantix), bupropion (Zyban), and nicotine patches, gum, and lozenges.

## Methods: participants, interventions, and outcomes

### Study setting {9}

All aspects of the study will take place in the county of Los Angeles, CA, in the USA. The implementation of smoking services will be delivered in eighteen outpatient clinics, eleven from LACDHS operated outpatient primary care clinics and seven from LACDMH-operated community mental health clinics. As part of the study, fifteen of the eighteen clinics will provide smoking cessation services and two clinics will continue to offer treatment as usual, without enhanced smoking cessation services. Randomization will be used to determine which clinics will provide smoking cessation services. Participating outpatient clinics from LACDHS are (1) Martin Luther King, Jr. Outpatient Center (2); LAC + USC Medical Center (3); Edward R. Roybal Comprehensive Health Center (4); Harbor-UCLA Medical Center – General Internal Medicine (5); Harbor-UCLA Medical Center – Family Medicine (6); Mid Valley Comprehensive Health Center (7); Long Beach Comprehensive Health Center (8); High Desert Regional Health Center (9); Hubert H. Humphrey Comprehensive Health Center (10); Olive View – UCLA Medical Center; and (11) H. Claude Hudson Comprehensive Health Center. Participating outpatient clinics from LACDMH are (1) East San Gabriel Valley Health Center (2); Harbor – UCLA Mental Health Center (3); South Bay Mental Health Center (4); West Central Family Mental Health Center (5); West Valley Mental Health Center (6); Arcadia MHC; and (7) American Indian Counseling Center.

### Eligibility criteria {10}

Inclusion criteria for participants are as follows: (1) be 18 years old or older, (2) smoke three or more cigarettes or cigars per day, (3) have thought about stopping smoking, and (4) be enrolled in care at either LACDHS and/or LACDMH. Participants who use only electronic cigarettes, hookah, or other types of tobacco/nicotine will not be allowed in the study if they do not meet all inclusion criteria. Cigarette and cigar smokers who also use electronic cigarettes, hookah, or other types of tobacco/nicotine will be allowed in the study.

Exclusion criteria for participants are as follows: (1) under 18 years, (2) smoke less than three cigarettes or cigars per day, (3) not interested in stopping cigarette smoking, and (4) not enrolled in care at either LACDHS and/or LACDMH.

Based on our preliminary data and analysis, a significant number of dropouts are anticipated. An estimated 50% of enrolled participants will drop out. Since this is a pragmatic real-world trial, we accept the risk of a significant dropout rate. Furthermore, given that this is an implementation trial, study participants can continue to participate in the research assessments even if they do not participate in clinical services. A patient is considered a dropout if they report no longer wanting to participate in the research assessments or cannot be reached to complete research assessments.

### Who will take informed consent? {26a}

At the first interview and prior to conducting any research-related procedures, the clinical coordinator will discuss the written informed consent and outline the study procedures. Once the participant has asked questions and has a clear understanding of the study, the participant will sign the consent form and will be enrolled into the study.

### Additional consent provisions for collection and use of participant data and biological specimens {26b}

Not applicable as no additional participant data and biological specimens were collected as part of this trial.

### Interventions

#### Explanation for the choice of comparators {6b}

This is a cluster-randomized comparative effectiveness trial that compares intervention clinics offering smoking cessation group counseling and medication management integrated into primary care and community mental health clinics with TAU clinics offering information about the CA Smoker’s Helpline and informal provider counseling.

#### Intervention description {11a}

Each clinical site in LACDHS is part of a cluster, and there will be a 5:1 ratio in the assignment of both LACDHS and LACDMH clinics assigned to the intervention as compared with TAU. The smoking cessation intervention will be a program embedded in the participating clinics and will include (1) a weekly 60–75-min CBT smoking cessation counseling group which will be led by facilitators that have been trained through the Kick Ash program and may be a nurse, pharmacist, clinician, SUD counselor, behavioral health clinician, or counselor and (2) smoking cessation medication that will be prescribed by a psychiatrist, pharmacist, or primary care doctor licensed clinician. Participants will be offered 6 weekly consecutive groups focusing on gaining necessary skills to aid in the decrease or total cessation of smoking behaviors. Those attending the groups will gain alternative coping strategies, relapse prevention strategies, and tools and resources to assist with reaching smoking cessation. Regarding the counseling group, participants will be able to repeat the groups willingly as needed until total smoking cessation is obtained. Smoking cessation medications will include varenicline (Chantix), bupropion (Zyban), nicotine patches, gum, and lozenges. Participants prescribed varenicline will be prescribed 0.5 mg of varenicline once daily for 3 days, then 0.5 mg twice daily for the next 4 days, then 1 mg twice daily thereafter. Those prescribed bupropion (Zyban) SR 150mg will be offered and, if accepted, prescribed once daily for 1 week, followed by bupropion SR 300 mg once daily thereafter. Participants prescribed NRT will be prescribed the standard prescription for nicotine NRT which includes a transdermal patch at a dose that approximates their current average mg of nicotine intake from tobacco products. Nicotine gums or lozenges (in either 2 mg or 4 mg strength) will also be offered and can be used as needed when experiencing cravings to smoke. TAU offers information about the California Smoker’s Helpline and usual provider and clinician counseling. Participants in TAU will be asked at each data collection point (baseline and 3, 6, and 12 months) about any smoking cessation services received including prescribed and over-the-counter smoking cessation medication.

#### Criteria for discontinuing or modifying allocated interventions {11b}

The clinician or pharmacist will discuss with participants in detail the individual side effects associated with the smoking cessation medication to help the participant decide whether or not to take the medication. During this time, participants will provide the clinician or pharmacist with an accurate smoking and medical history to ensure the prescribed medication is safe for them. At each in-person study interview, the researcher will complete brief medical and mental health assessments. To note, in-person study interviews shifted to telephone interviews in response to COVID-19. Participants who experience any side effects can choose to meet with their healthcare provider to help them resolve the side effects by either discontinuing medication, modifying medication dosage, or other responses left to the discretion of the healthcare provider.

#### Strategies to improve adherence to interventions {11c}

To assist with adherence to intervention protocols, participants will be offered medications using a shared decision framework. Shared decision-making is an established medical practice where patients are invited to participate in selecting the treatment strategy that they would like to obtain [11, 12]. This has been associated with improved adherence to medication treatments. Participants will choose their own preferred treatment, pending any contraindications, during the 15-min medication management visit with a prescribing clinician. The shared decision-making medication protocol will permit flexible prescribing, such that participants can establish a quit date further than a week from initiation of smoking cessation medication. Additionally, participants can continue medication usage even if they continue actively smoking, provided that the clinician identifies ongoing clinical benefits to the smoking cessation medication treatments. To further assist with interview adherence, interviews will take place at the participant’s primary care clinic or the site they usually obtain county-provided services. Participants who are assigned to clinics that provide smoking cessation services will also be asked about the frequency of their participation in the smoking cessation services during the 12-month period.

### Relevant concomitant care permitted or prohibited during the trial {11d}

Concomitant medications that make it unsafe for a participant to be prescribed smoking cessation medication will be decided by the participant’s healthcare provider. All other concomitant care will be allowed.

### Provisions for post-trial care {30}

There are no provisions for ancillary and post-trial care. As the risks of the study are minimal, there are no plans to compensate those who suffer harm from trial participation. If a participant experiences an adverse event to the medical care received at a county site, they will be instructed to follow up with their healthcare provider.

### Outcomes {12}

The primary outcome of the study is higher rates of smoking cessation in the intervention sites as compared with matched LAC-operated setting non-intervention sites. Exploratory analysis will consider intervention effects on the smoking rate, such as smoking reductions. To measure rates of smoking cessation, study participants will complete two objective measures collected at four time points at 3-month intervals within a 12-month period. Analysis will be at each time point compared to baseline. The objective measures include a carbon monoxide breathalyzer (piCO+Smokerlyzer) and a urinary rapid dipstick cotinine test (NicCheck) before March 2020. However, these measures were temporarily discontinued in response to COVID-19 when data collection shifted from in-person to telephone collection.

The study will evaluate the (1) penetrance of smoking cessation medications and counseling services, at intervention and non-intervention sites and between mental health and primary care clinics; (2) self-reported cigarette use, smoking urges, number of quit attempts, changes in self-reported anxiety, stress, quality of life, and participant satisfaction; (3) and the dose effect of medication and counseling services on smoking cessation.

### Participant timeline {13}

**Fig. 1:**
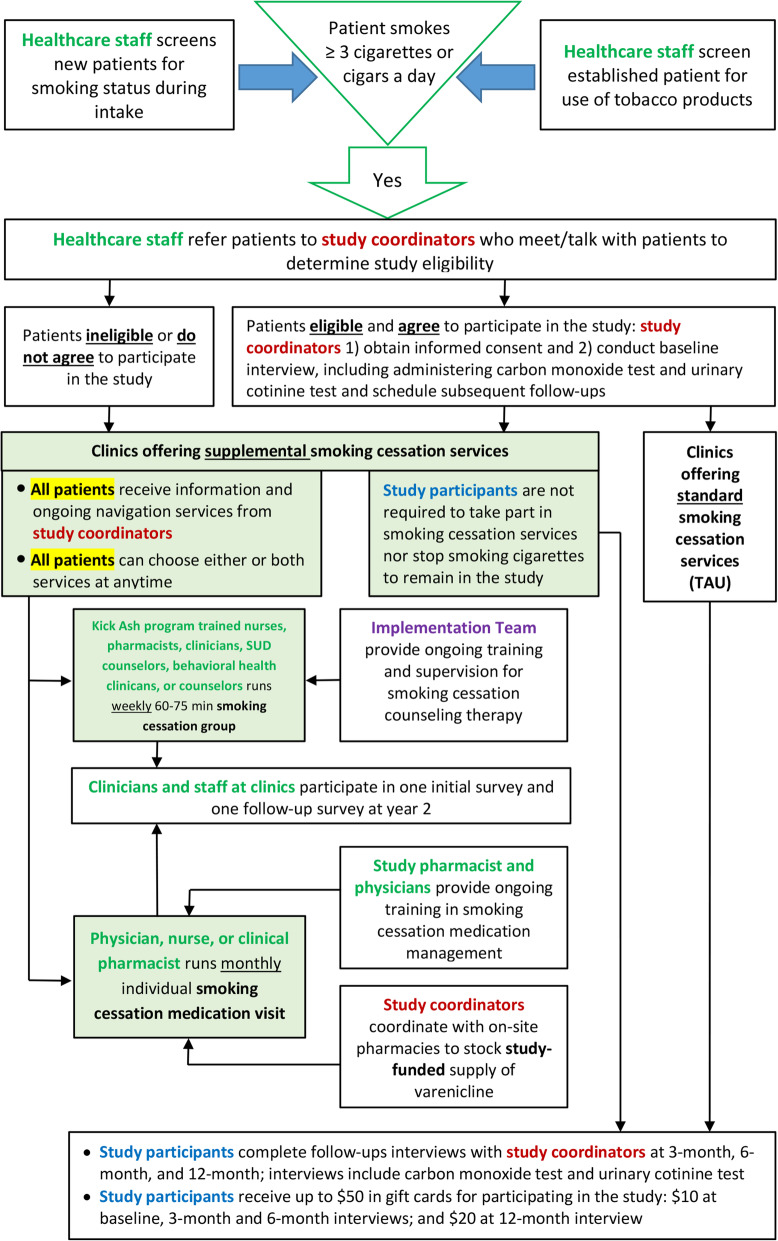
Flow diagram illustrating participant’s timeline through the trial

The study design begins with implementation and delivery of the smoking cessation services, followed by healthcare staff surveys, study participant recruitment by the healthcare staff, enrollment into the study and smoking cessation services navigation by the research team for participants enrolled in clinics that will provide smoking cessation services, and participant follow-ups administered by the research team. See Fig. 1 for the study flowchart.

### Sample size {14}

Prior to the beginning of the study, a formal power analysis was done. The plan is to enroll 1000 smokers in 14 clinical sites where specialty tobacco use disorder services (STUDS) are being offered and 200 smokers in 3 clinics in the TAU clinical sites and expect a dropout rate of 50%. Thus, a cluster-randomized study design was envisioned. If we assume that the intervention sites achieve a 25% success rate at achieving abstinence and the TAU group a 5% success rate, then with a reasonably sized intra-cluster correlation coefficient of 0.05, the power to detect a difference in success rate is 99.4%. This assumes a two-sided test of proportions (using a large sample approximation and the corresponding cluster adjustment) with a significance level of 5%.

### Recruitment {15}

All patients currently enrolled at LACDHS and LACDMH sites who meet inclusion criteria are eligible to participate. Participants will be recruited by healthcare providers and staff at LACDHS and LACDMH. An invitation to participate in the study will be offered by LACDHS and LACDMH healthcare providers to all LACDHS and LACDMH enrolled clients, regardless of concurrent medical or mental health issues, through initial evaluations at intake appointments and through regular healthcare appointments with existing clients. To assist with reaching the target sample size, recruitment efforts will extend to individuals on Medi-Cal, My Healthy Way LA beneficiaries, and individuals without insurance coverage. LACDHS and LACDMH healthcare providers will refer patients interested in the study to the research staff located at that site. Furthermore, healthcare providers will invite potential study participants through flyers, video presentations, and distribution of study referral information. Enrolled LACDHS and LACDMH clients can also self-refer at any time and do not need to be referred by their healthcare provider. LACDHS and LACDMH healthcare providers will refer interested smokers to the research team through the LACDHS and LACDMH electronic records, by telephone or email. Following the telephone screening, research coordinators will invite those meeting inclusion criteria to sign an informed consent and to enroll in the study, streamlining recruitment efforts.

## Assignment of interventions: allocation

### Sequence generation {16a}

Block randomization will be by cluster using the program calculator.net. Each clinical site is a cluster, and there will be a 5:1 ratio in the assignment of *both* LACDHS and LACDMH clinics assigned to the intervention arm (offering STUDS) as compared with the TAU arm. A total of 18 study sites will be randomized on a 5:1 intervention to TAU ratio basis, with three TAU sites and fifteen intervention sites split as follows: 11 primary care clinics (9 intervention, 2 TAU) and 7 mental health clinics (6 interventions, 1 TAU).

### Concealment mechanism {16b}

There is no concealment.

### Implementation {16c}

Statistician Martin L. Lee, PhD, will perform the randomization at a clinic level using a computer program. The study is a cluster-randomized design protocol. Individual clinics will be assigned to the intervention and TAU on a 5:1 ratio. Study participants empanelled or otherwise being treated by clinicians at TAU clinics are therefore considered to be in the TAU arm, and study participants who receive care from intervention sites are in the intervention arm. Clinic staff and study coordinators will notify clinics of their assignments. Following the eligibility check and informed consent, the research team will enroll participants into either clinics that provide smoking cessation services or TAU clinics. The sites will be identified by a rotating code, so participants cannot learn which sites are TAU.

## Assignment of interventions: blinding

### Who will be blinded {17a}

There is no blinding.

### Procedure for unblinding if needed {17b}

There is no need for unblinding.

## Data collection and management

### Plans for assessment and collection of outcomes {18a}

Data will be collected in-person by research interviewers at the participant’s primary care clinic or site associated with where they usually obtain county-provided services. Research interviewers will complete brief medical and mental health assessments, including smoking status. Study measures will be administered on a tablet computer and captured in real time using Qualtrics, a secure, direct data entry method HIPAA-compliant Web-based survey platform. Each data collection point will take approximately 30–45 min. The following interviews and self-report measures will be administered during the baseline, 3-month, 6-month, and 12-month in-person visits: (1) Beck Anxiety Inventory (BAI), a self-report assessment to measure anxiety symptomatology [13]; (2) Beck Depression Inventory, Revised (BDI-II), a widely used measure in psychological research and clinical practice of depressive symptomology [14]; (3) Fagerstrom Test for Nicotine Dependence (FTND), a widely used study measure of nicotine dependence [15]; (4) Minnesota Nicotine Withdrawal Scale (MNWS) to measure symptoms of craving for tobacco, irritability, anxiety, difficulty concentrating, and restlessness after smoking cessation [16]; (5) Wisconsin Predicting Patient’s Relapse WI-Prepare, a scale that predicts smoking cessation outcomes [17]; (6) 30-day Timeline Followback Assessment (TLFB) measures quantity and frequency of smoking [18]; (7) Daily Smoking Log to assess smoking frequency in the 7 days prior to group meeting; (8) Questionnaire of Smoking Urges-Brief (QSU-Brief) designed to measure research participants’ level of craving and urges to smoke cigarettes assessment [19]; (9) Patient Satisfaction Questionnaire Short Form (PSQ-18) assessing satisfaction with medical care [20]; (10) World Health Organization Quality of Life-Brief (WHOQOL-BREF), a self-report assessment to measure the quality of life, with subscales, which has been extensively validated in studies worldwide ; and (11) The Quit Smoking Measure will consider study participants to have “quit” smoking cigarettes during the study if they self-report smoking no cigarettes within the last 7 days. We initially planned that this would be corroborated by exhaled carbon monoxide level of <5 ppm (parts per million). CO breathalyzer (piCO+Smokerlyzer) test and rapid dipstick cotinine test (NicCheck) were administered at each in-person study visit, but breathalyzer and urine testing were temporarily discontinued in March 2020 due to the pandemic. See Table 1 for procedures and measures administered at each study visit.

**Table 1:** Procedures and measures administered at each study visit

Month	0	3	6	12
Study visit	1	2	3	4
Informed consent	X			
Medical assessments	X	X	X	X
Behavioral Measures Battery	X	X	X	X
Weekly Cigarette Log	X	X	X	X
Patient Satisfaction Questionnaire Short Form (PSQ-18)	X	X	X	X

### Plans to promote participant retention and complete follow-up {18b}

To promote participant retention and respect the participants’ time during the study, participants will be compensated up to $50. Participants will receive a $10 gift card for the baseline, 3-month, and 6-month interview. At the final interview (12-month), participants will receive a $20 gift card. Compensation will be in the form of gift cards to a local store and will be made at the end of each of the four interviews. Additionally, all interviews will take place at the participant’s primary care clinic, or the site associated with where they usually obtain county-provided services, curbing possible inconveniences or transportation barriers to each study visit. To minimize missing data for those elements manually transcribed into Qualtrics, we will use that application’s data type restrictions to help enforce entry of required data elements.

### Data management {19}

The research team will store all study-related data in an electronic data capture (EDC) eCRF system (Qualtrics). Qualtrics is a secure, direct data entry method that is HIPAA compliant. This system is password protected and only accessible to the research team. Physician data includes paper source documents. The research team will provide pencil-and-paper individual surveys to physicians (*Clinical Staff Questionnaire – Clinical Survey*). Other selected clinic care team staff will be asked to complete the paper-and-pencil survey by their clinic administrator. The paper source documents will also be available online using the eCRF system, Qualtrics. Data will be transcribed from source documentation directly into a statistical program such as Statistical Analysis System (SAS). Clinic data will consist of data from the Online Real-time Centralized Health Information Database (ORCHID) at LACDHS and the Integrated Behavioral Health Information System (IBHIS) at LACDMH about relevant care processes at the participating clinics, both those randomized to offer the group session and those randomized to not offer the group session. Such data will be aggregated at the clinic level and provided to the research team by LACDHS/DMH staff based on the availability and capacity of LACDHS/DMH. Regulatory bodies such as the funding agency and/or Los Angeles County Department of Public Health Committee for the Protection of Human Subjects may have access to study data for the purpose of an audit to protect the rights and welfare of participants. Auditors are trained in the rules of confidentiality and will take the utmost care to keep study data confidential.

Identification numbers will be used to store the collected data, such as assessments. All study-related data will be stored in a secure, Web-based data capture system. The system will be password protected and only accessible to the research team. A cross-index of identifiable information will only be accessible to the research team to link participants to the identification numbers. Participants’ contact information and any other documents that contain names or any identifying information collected on paper will be scanned and saved to a HIPAA-compliant document storage system on ORCHID or IBHIS in accordance with the participants’ primary care clinic. The documents will then be confidentially destroyed. Files which contain any type of identifying information will be confidentially destroyed 5 years after the end of the study.

### Confidentiality {27}

Any information that is obtained in connection with this study that can identify participants will remain strictly confidential. The research team is trained in the rules of confidentiality as required by law and it is the research team’s responsibility to ensure that participant confidentiality is protected at all times. However, if the research team is required by law to disclose confidential information to the appropriate authorities to protect participant’s well-being and/or the well-being of others, the team will do so without participant’s consent. Additionally, study data or information that is published and discussed in conferences or in presentations will not include anything that could reveal participants’ identity.

To protect confidentiality during the smoking cessation counseling group, the facilitator leading the group will stress the importance of confidentiality among group attendees, emphasizing that attendees should not mention anything said in the group to anyone outside of the group. Additionally, the participants will share information during the counseling group at their own discretion. Clinic staff or participants’ LACDHS/LACDMH healthcare provider may eventually know that they are participating in the study. However, they do not have access to nor will be given any information the participants provide to us as part of the study.

Authorized representatives from the funding agency and/or from the Los Angeles County Department of Public Health Committee for the Protection of Human Subjects may ask to access the study data for the purposes of an audit. Auditors are trained in the rules of confidentiality and will take the utmost care to keep study data confidential.

### Plans for collection, laboratory evaluation, and storage of biological specimens for genetic or molecular analysis in this trial/future use {33}

Not applicable as we will not be collecting or storing biological specimens.

## Statistical methods

### Statistical methods for primary and secondary outcomes {20a}

Final analyses will begin in year 4 at quarters 2 and 3. Data analysis will utilize an intention-to-treat (ITT) approach as the primary analysis. An additional set of analyses will use a per-protocol (PP) paradigm that will only use subjects who attend at least 3, out of four visits, including month 12. Descriptive statistics will include number, percent, and mode for categorical data (gender, race/ethnicity, site, LACDHS/LACDMH, number of visits). Continuous variables of clinical characteristics (number of cigarettes smoked, number of years smoked, Fagerstrom score) and Behavioral Measures Battery will be used to describe the study population. Non-normal distribution will be transformed to satisfy the normality assumption for the parametric statistical tests. Scale variables will be presented as min, max, and median. Graphical presentations (histograms, boxplots, and spaghetti plots) will be used to facilitate the visualization of data and the time trend of endpoints and baseline variables. All statistical analyses will be performed using SAS 9.2 and a *P*-value <0.05 will be considered statistically significant.

For model 1, we will perform an analysis of covariance with the baseline value (site, racial-ethnic group, number of visits, age, weight, smoking status, and survey scores) as the covariate and compare the value at the end of the study. To evaluate the change over time between the groups that incorporates all the measures collected over the timeframe of the study, then a two-way repeated measures analysis of variance model will be used. For model 2, a multiple logistic regression model would be built using independent variables such as treatment group and pre-determined baseline covariates (including study site). We posit that using a covariate-adjusted model to evaluate outcome provides a sensitivity analysis for the primary results of a simple comparison of the achievement of smoking cessation. For both models, the analyses will incorporate the cluster-randomized design into the model using an appropriately chosen variance-covariance structure (up to the use of an unspecified structure). In addition, the models will include a term for LACDHS versus LACDMH sites and their interaction with the intervention group.

### Interim analyses {21b}

Preliminary data analyses will occur over two quarters beginning at quarter 4 of year 2. The interim analysis will examine the number of cigarettes smoked per day per participant and the number of subjects who have stopped smoking.

### Methods for additional analyses (e.g., subgroup analyses) {20b}

As a supplemental set of analyses, we will examine the questionnaires and their ability to predict smoking cessation. The questionnaires will be used as originally designed such that psychometric data for each questionnaire will inform their scoring and interpretation.

### Methods in analysis to handle protocol non-adherence and any statistical methods to handle missing data {20c}

The primary analysis will use the intent-to-treat approach (ITT) which requires all randomized participants to be included in the statistical analysis and use the last value carried forward method to account for missing data, which tends to be conservative. Thus, if a patient only comes once, their smoking status and other characteristics will be that of baseline measurement and no change in the values with time will be noted.

### Plans to give access to the full protocol, participant-level data, and statistical code {31c}

We will provide the protocol, participant-level data, and statistical code upon request.

## Oversight and monitoring

### Composition of the coordinating center and trial steering committee {5d}

The research team will provide oversight for the conduct of the research study. The research team will record changes in policy that occur over the study period and that relate to the general care environment to LACDHS/DMH clinics. Additionally, the research team will record issues and barriers that arise over the study period as well as changes to workflow that the team makes during implementation. Periodic study team meetings will serve as the source of this information on implementation context, barriers, and changes. The DPH IRB and Department of Mental Health HSRC reviewed the protocol and consent and recommended changes that were incorporated into both. The IRB deemed that a data safety monitoring board was unnecessary as the study is low risk. Furthermore, the study is an implementation trial using already established treatments, and the primary aims are not assessing if treatment work but whether the treatments work in real-world contexts. Thus, a community advisory board was assembled composing of community members and staff members of the participating clinics served as the study’s independent steering committee.

### Composition of the data monitoring committee, its role, and reporting structure {21a}

Because the study has minimal risk, the DPH IRB and HSRC determine that no data safety monitoring board (DSMB) or data safety monitoring plan (DSMP) is needed.

### Adverse event reporting and harms {22}

Although the study has minimal risks and we do not expect any adverse events, we will notify the Department of Public Health (DPH) Institutional Review Board (IRB) and Department of Mental Health HSRC should they occur. During the consenting process, the study procedures will be explained to participants to help reduce any risks. The study coordinators will collect information from participants, including whether they are having an adverse experience with medications, but because the medication management is managed by the clinic and not as a research intervention, we direct participants to contact their prescribing clinicians and can facilitate patients making that connection. The only adverse events that would be collected as part of the study itself are related to the research procedures such as problems with the survey administration, CO testing, and urine testing. Collected data will not include adverse events with the care patients are receiving at the clinics. Albeit not collected, the most frequent adverse event of varenicline includes nausea (25%), of bupropion includes insomnia (12%), and of nicotine patch includes abnormal dreams (12%) [21].

### Frequency and plans for auditing trial conduct {23}

We do not envision the need for an audit. It is possible, the DPH IRB or the Department of Mental Health HSRC may audit our study.

### Plans for communicating important protocol amendments to relevant parties (e.g., trial participants, ethical committees) {25}

The study has minimal risks and explaining the study during the consenting process will help reduce the risks. We do not expect any adverse events, but we will notify the DPH IRB and HSRC should they occur.

### Dissemination plans {31a}

Mrs. Norma Mtume, our community engagement specialist, will help to ensure that the findings are communicated in the most appropriate way to the desired audiences. She will be available for presentations at professional and community forums, conferences, and workshops. Furthermore, the project team will disseminate information about the project itself and later our research findings at local and national conferences. We will extend our reach to students and other trainees in the health professions field so that future health professionals are aware of findings. Finally, we will publish our findings in peer-reviewed scientific publications and local community publications or other outlets in order that the community may learn of our study outcomes. Mrs. Mtume had such an article published in the Los Angeles Sentinel in January 2018 that highlighted TRDRP research being conducted in South Los Angeles. She co-authored a poster presentation regarding embedding community stakeholders in T1-T2 research teams which was presented at professional conferences.

## Discussion

Smoking cessation best practices include coordinated medication and behavioral treatments. Specifically, medication such as nicotine replacement therapy, bupropion, and varenicline have been proven to be effective in assisting with smoking cessation. However, our preliminary data found that only 21.3% of identified smokers received some sort of intervention in DHS and DMH clinics. For these reasons, the present 5-year implementation study will offer group counseling as well as medication for smoking cessation services delivered in LACDHS and LACDMH clinics compared to matched LAC-operated sites with treatment as usual.

The study represents a collaboration of three large departments within the Los Angeles County Health Agency that together treat the majority of Medi-Cal beneficiaries in Los Angeles County. Individuals served by the health departments within the Los Angeles County Agency are disproportionately affected by tobacco use disorder, leading to a significant treatment gap in these public-funded medical and mental health settings. Therefore, it is important to set up a comprehensive smoking cessation clinic in DMH and DHS clinics that will offer counseling as well as medication for smoking cessation, as the present study does. Understanding the effectiveness and feasibility of smoking cessation services delivered in LACDHS and LACDMH clinics in comparison to treatment as usual will be useful in evaluating future implementation and potentially bridging the treatment gap in these publicly funded medical and mental health settings.

Although there are strengths of the study, there are notable limitations. In response to the COVID-19 pandemic, the breathalyzer and urine testing were temporarily suspended in March 2020 and not anticipated resuming in-person visits. The CO breathalyzer and urinary cotinine test was originally going to be used to corroborate participants’ abstinence from smoking cigarettes for seven consecutive days and assess levels of nicotine in the body. Furthermore, the COVID-19 pandemic has required study staff to shift from in-person group visits to individual visits done by telephone and consenting shifted to be completed by mail. Although the COVID-19 pandemic has required adjustments, the integrity of the study has continued to be upheld.

The success of completing the study will provide information on the effectiveness of what smoking cessation services or combination of services are helpful to individuals seeking smoking cessation. In addition, the study findings will provide information to outpatient clinics on the feasibility and procedures on setting up smoking cessation services as part of core services. Most notably, the services are delivered by LACDHS and LACDMH clinicians in LAC-operated settings; therefore, if found effective, the services will continue after study completion.

## Trial status

Recruitment began on October 1, 2019. Recruitment and follow-up visits are set to end on June 3, 2022. The current protocol is version 7 created on January 16, 2021.

## Supplementary Information

**Additional file 1.** The English versions of the informed consent.

## Abbreviations

BAI: Beck Anxiety Inventory
BDI-II: Beck Depression Inventory, Revised
CBT: Cognitive Behavioral Therapy
CO: Carbon Monoxide Breathalyzer
DHS: Department of Health Services
DMH: Department of Mental Health
DPH: Department of Public Health
DSMB: Data Safety Monitoring Board
DSMP: Data Safety Monitoring Plan
EDC: Electronic data capture
FTND: Fagerstrom Test for Nicotine Dependence
GED: General Education Development
HIPPA: Health Insurance Portability & Accountability Act
HSRC: Human Subjects Research Committee
IBHIS: Integrated Behavioral Health Information Database
ITT: Intent to treat
LAC: Los Angeles County
LACDHS: Los Angeles County Department of Health Services
LACDMH: Los Angeles County Department of Mental Health
MHAD: Mental Health and Addictive Disorders
MNWS: Minnesota Nicotine Withdrawal Scale
NRT: Nicotine replacement therapy
ORCHID: Online Real-time Centralized Health Information Database
PCMH: Patient-Centered Medical Homes
PP: Per protocol
PPM: Parts per million
PSQ: Patient Satisfaction Questionnaire
QSU-Brief: Questionnaire of Smoking Urges-Brief
SAS: Statistical Analysis System
STUDS: Specialty Tobacco Use Disorder Services
SUD: Substance use disorder
TAU: Treatment as usual
TLFB: Timeline Followback Assessment
TRDRP: Tobacco Related Disease Research Program
TUD: Tobacco use disorder
WHOQOL-BREF: World Health Organization Quality of Life-Brief
