# Supplementary material for: Embedding comprehensive smoking cessation programs into community clinics: study protocol for a cluster-randomized controlled trial
Source: Trials. 2022 Feb 3;23:109. doi: 10.1186/s13063-022-06023-3 (PMC8811740; doi:10.1186/s13063-022-06023-3)
Supplement: Supplementary file 1 — Additional file 1. The English versions of the informed consent. [file 13063_2022_6023_MOESM1_ESM.pdf]

CONSENT TO PARTICIPATE IN A RESEARCH STUDY

Embedding comprehensive smoking cessation programs into community clinics

(For Clinics Offering Supplemental Smoking Cessation Services)

Why am I being asked to volunteer?

You are being asked to volunteer for a research study conducted by Friends Research Institute under the direction of Drs. Theodore Friedman and Brian Hurley. You are eligible to participate in this study because you are 18 years old or older, you smoke three or more cigarettes or cigars a day, you have thought about stopping cigarette/cigar smoking, and you are receiving care at a Los Angeles County Department of Health Services (LACDHS) and/or Department of Mental Health (LACDMH) clinic.

This study is funded by the Tobacco Related Disease Research Program (TRDRP). The study will evaluate smoking cessation, which means stopping or ending smoking. There are 17 outpatient clinics in Los Angeles County that are participating in this study, 11 from LACDHS and 6 from LACDMH. This clinic will provide services which will include a weekly smoking cessation counseling group and smoking cessation medications. These services will be provided at no charge to anyone who receives services at LACDHS and/or LACDMH outpatient clinics.

Our aim is to enroll 1200 cigarette/cigar smokers into this study from the 17 clinics.

Your participation in this study is entirely voluntary. This consent form explains the study in detail. The Smoking Cessation Navigator will go over this form with you. Feel free to ask questions about anything you do not understand before deciding whether to participate. Please take as much time as you need to read this form.

In addition, your health care provider may be an investigator of this study protocol, and as an investigator, is interested in both your clinical welfare and in the conduct of this study. Before entering this study or at any time during the study, you may ask for a second opinion about your care from another doctor who is in no way associated with this study. You are not under any obligation to participate in any research study offered by your physician.

You do not need to stop smoking cigarettes/cigars to be in the study. You will remain in the study no matter what your smoking status.

What is the purpose of the study?

The purpose of the study is to collect information on what types of resources and efforts are needed in order to provide smoking cessation services within county-operated outpatient clinics. In addition, the study will evaluate smoking cessation rates across the 17 clinics.

Smoking cessation services at this Clinic

This clinic will provide a weekly, 60-75 minute smoking cessation counseling group and smoking cessation medications at no cost to all patients who receive services at this clinic and affiliated clinics.

The smoking cessation medications include varenicline (Chantix), bupropion (Zyban) and nicotine patches, gum, and lozenges. Any patient interested in receiving a prescription for any of these medications will meet with the prescribing clinician to help choose the best prescription option. The medication will be provided at no cost through either this clinic's pharmacy or through the patient's usual retail pharmacy.

- These services are available to all patients at this clinic and affiliated clinics.
- You do not need to be in this study to take advantage of these services.
- You do not need to participate in these services to be in the study.

What procedures are involved?

If you volunteer to participate in this study, you will be asked to meet with a Smoking Cessation Navigator four times in the next 12 months either in-person or by telephone for approximately 45 minutes each time. At each meeting you will be asked to:

- Complete brief medical and mental health assessments which will ask about your overall physical and mental health, including your smoking status.
- Complete a carbon monoxide test (CO) which involves blowing into a CO breathalyzer which measures the level of carbon monoxide in your lungs. This test measures if and how much you are smoking and will be completed only during an in-person meeting with the Smoking Cessation Navigator.
- Provide a urine sample which will be tested for cotinine (a substance related to nicotine that determines if and how much you are smoking). The urine collected for this study will only be tested for cotinine and no other substances and will be completed only during an in-person meeting with the Smoking Cessation Navigator.
- Provide contact information that will help us to keep in touch with you for your next scheduled meetings. If you miss a scheduled meeting you will still remain in the study.

In-person meetings will take place at your clinic and may be scheduled on the same day as another scheduled appointment at your clinic for your convenience.

If you agree to participate in the study and you decide to attend the smoking cessation counseling group, we will ask you to:

- Complete a daily calendar where you will write down the number of cigarettes/cigars you smoked in the week before the group meeting.
- Complete a CO breathalyzer test before or after the group meeting.

We will ask you to review and sign or verbally agree to the Health Insurance Portability and Accountability Act (HIPAA) form in which you give permission to LACDHS/LACDMH to provide us limited information from your medical record. The information will include:

- Your name, telephone numbers and email addresses so that we are sure to stay in touch with you in the next 12 months.
- Your attendance in the smoking cessation counseling group (if you choose to attend).
- Your smoking cessation medication prescription (if you choose to receive a prescription).

If you do not sign or verbally agree to the HIPAA form, you cannot be in the study.

What are the potential risks and discomforts?

There is a very small chance that you may experience the following scenarios:

- If you attend the smoking cessation counseling group, there is a small chance that what you say in the group may not be kept confidential by other group members. To minimize this, the counselor/clinician who leads the group will stress the importance of confidentiality among group attendees and that attendees should not mention anything said in the group to anyone outside the group. In addition, it is up to you to choose what you want to share in the group.
- There is a small possibility that you may feel uncomfortable with some of the questions asked during a scheduled meeting with the navigator. If you feel uncomfortable, you can choose not to answer the question, take a break or discontinue the meeting. You can also choose to have the navigator call a clinic staff member to make sure you have someone to talk to about the question or the meeting.
- If you reduce or stop smoking cigarettes/cigars, you may experience common symptoms associated with nicotine withdrawal which includes headaches, depressive symptoms, irritability, trouble sleeping, weight gain and increased cravings for cigarettes/cigars. If you experience any of these symptoms, you can choose to meet with your health care provider to help you resolve any symptoms.
- There are individual side effects associated with the smoking cessation medications which the clinician/pharmacist will discuss with you in detail which will help you decide whether or not you want to take the medication. If you choose to take the smoking cessation medication and you experience any side effects, you can choose to meet with your health care provider to help you resolve any side effects.
- There is a very small chance that information you provide as part of the study may not remain confidential. We have set up several safeguards to protect your privacy so that chance of this happening is very small (see "What about privacy and confidentiality?" section below).
- Staff at this clinic or your LACDHS/LACDMH health care provider may eventually know that you are participating in this study. However, they do not have access to nor will be given any information you provide to us as part of the study.

What are the potential benefits to me and/or to society?

By participating in the smoking cessation group visits, you may not only successfully quit smoking, but you may also prevent or reverse further health problems. The benefit of participating in the research related to collecting your data or the surveys/discussion groups is minimal for you. Findings from this study may provide information to outpatient clinics about how to set up smoking cessation services as part of their core services. In addition, the data may also provide information about what smoking cessation service or combination of services are helpful to people who want to stop smoking cigarettes/cigars in the future.

How much will I get paid to participate in the study?

You can earn up to $50 for participating in the study. You will receive payment for each of the 4 meetings with the Smoking Cessation Navigator where you complete the assessment, your contact information and the CO breathalyzer and urine sample when applicable. Payments will be as follows:

- $10 for the 1st meeting
- $10 for the 2nd meeting
- $10 for the 3rd meeting
- $20 for the 4th meeting

You will receive payment at the end of each in-person meeting. Payment for meetings conducted over the telephone will be mailed to the address you provide. Your payment will be a gift card to either Target or Food for Less.

Are there any costs to me for participating in this study?

No, you will not pay anything to participate in this study.

What about privacy and confidentiality?

Any information that is obtained in connection with this study will remain strictly confidential. However, if we are required by law to disclose confidential information to the appropriate authorities in order to protect your well-being and/or the well-being of others, we will do so without your consent.

We will protect your privacy in the following ways:

- We will not use you name on any data collected at your meetings with the navigator, instead we will use an identification number to store your data.
- Any study data or information that is published, discussed in conferences or in presentations will never include anything that could reveal your identity.
- The research team will store all study related data in a secure, web-based database that is password protected and only accessible to the research team.
- Any other documents and audio recording (if applicable) that contains your name (for example this consent form and Health Insurance Portability and Accountability Act (HIPAA) form) will be securely stored and will be confidentially destroyed at the end of the study.

What other options are there?

If you choose not to participate in the study, you will still have access to the smoking cessation services offered at this clinic. The smoking cessation counseling group and the smoking cessation medications are available at no charge to anyone who receives services at LACDHS and/or LACDMH outpatient clinic.

Can I withdraw or be removed from the study?

Yes. Your participation in this study is VOLUNTARY. If you choose not to participate in the study your relationships with LACDHS and/or LACDMH will continue as usual. There is no penalty or loss of benefits to which you are otherwise entitled. If you decide to participate, you are free to withdraw your consent and discontinue participation at any time without prejudice to your future care at LACDHS and/or LACDMH.

The investigators also have the right to withdraw you from the study without your consent, if the circumstances require it. This would mean that you would no longer meet with the smoking cessation navigator. If you are withdrawn from the study, you can still attend the smoking cessation counseling group and obtain smoking cessation medication as these services are available at no charge to anyone who receives services at any LACDHS and/or LACDMH outpatient clinic.

What about any new information that may affect my decision to participate?

If researchers discover any significant new findings (either good or bad) during the course of the study we will provide you with a full explanation of the findings so that you can decide if you want to continue in the study. If you decide to continue in the study, we will ask you to sign an updated consent form which will include an explanation of the new findings.

Can I be physically injured by participating?

No. There is no known risk of physical injury to you in participating in the study. If you are injured as a direct result of research procedures you will receive medical treatment, however you or your insurance will be responsible for the cost.

Who should I contact if I have questions about the study?

If you have any questions or concerns about the study, please feel free to call the Smoking Cessation Navigators at (323) 238-5611 or (323) 553-0273, or the principal investigators: Dr. Friedman at (702) 608-6906 and Dr. Hurley at (323) 457-3675.

What are my rights as a research participant?

You may withdraw your consent at any time and discontinue participation without penalty. You are not waiving any legal claims, rights or remedies because of your participation in this research study. If you have questions regarding your rights as a research participant, you may contact the Los Angeles County Department of Public Health Committee for the Protection of Human Subjects at (213) 288-8675. They are located at 313 N. Figueroa St., Room 127 Los Angeles, CA 90012.

DMH CLIENTS: Who should I contact if I have questions or concerns about my usual care and my participation in this study?

Clients served by the Los Angeles County Department of Mental Health (LACDMH) directly-operated clinics or LE contractors with questions or concerns regarding the impact of their research activities on access to our quality of their usual care may contact the LACDMH Human Subjects Research Committee at (213) 639-6348.

SIGNATURE OR VERBAL CONSENT OF RESEARCH SUBJECT OR LEGALLY AUTHORIZED REPRESENTATIVE

I have read (or someone has read to me) and understand the information provided above. I have been given an opportunity to ask questions and all of my questions have been answered to my satisfaction. I understand that I am not giving up any of my legal rights in signing this consent form or by giving my verbal consent. I have received a copy of this consent form.

BY SIGNING THIS FORM OR BY GIVING MY VERBAL CONSENT, I WILLINGLY AGREE TO PARTICIPATE IN THE RESEARCH IT DESCRIBES.

____
Name of Participant (Say name and date for verbal consent)

____ __________________
Signature of Participant Date

SIGNATURE OR VERBAL CONSENT OF INDIVIDUAL OBTAINING CONSENT

I have explained the research to the subject or his/her legally authorized representative and answered all of his/her questions. I believe that he/she understands the information described in this form and freely consents to participate.

____
Name of Individual Obtaining Consent (Say name and date for verbal consent)

____ __________________
Signature of Individual Obtaining Consent Date

Form Valid For Enrollment From
12/10/20 to 01/23/21
Los Angeles County-Public Health
Institutional Review Board
